# Proline Increases Pigment Production to Improve Oxidative Stress Tolerance and Biocontrol Ability of *Metschnikowia citriensis*

**DOI:** 10.3389/fmicb.2019.01273

**Published:** 2019-06-11

**Authors:** Ye Liu, Lanhua Yi, Changqing Ruan, Shixiang Yao, Lili Deng, Kaifang Zeng

**Affiliations:** ^1^College of Food Science, Southwest University, Chongqing, China; ^2^Food Storage and Logistics Research Center, Southwest University, Chongqing, China

**Keywords:** proline, *Metschnikowia citriensis*, maroon pigment, intracellular iron content, apoptosis, biofilm formation

## Abstract

Utilizing antagonistic yeasts is a promising approach for managing postharvest decay of fruits. However, it is well established that various severe stresses encountered in the environment and production process cause the intracellular reactive oxygen species (ROS) accumulation in yeast cells, resulting in cell damage and loss of vitality. Here, proline has been shown to function as a cell protectant and inducer of biofilm formation able to increase the oxidative stress tolerance and the biocontrol ability of the antagonistic yeast *Metschnikowia citriensis*. Addition of proline to *M. citriensis* cells induced a significant rise in superoxide dismutase (SOD) and catalase (CAT) activity in the early and late stages of oxidative stress, respectively, and increased the maroon pigment production that directly reduced intracellular iron content and indirectly diminished intracellular ROS levels and thus inhibited ROS- and iron-induced apoptosis. Treating cells with iron chelator tropolone yielded similar results. Pigment production induced by proline also enhanced the capability of biofilm formation of *M. citriensis*. These results suggested an important role for pigment of *M. citriensis* in response to oxidative stress. The abilities of proline to scavenge intracellular ROS and inhibit apoptosis, increase pigment production, and promote biofilm formation contribute to the improvements in oxidative stress tolerance and biocontrol efficacy of *M. citriensis*.

## Introduction

Postharvest fruits decay caused by fungal pathogens results in a large amount of economic losses and possible occurrence of mycotoxin contamination (Mahunu et al., 2016; Zhang et al., 2018). Utilizing various disease control agents to reduce postharvest losses is the most frugal ways to improve food security and nutritional value (Zhang et al., 2018). Based on the severe environmental pollution, human poisoning, and various diseases brought by the massive use of chemicals, biologicals (including biocontrol microbes) appear to be significant tools for the diseases control (Hazell and Wood, 2008; Aktar et al., 2009; Pandin et al., 2017; Yang et al., 2017; Ab Rahman et al., 2018; Yan et al., 2018).

Over the past 30 years, isolating wild species of antagonistic yeasts has become a research topic that receives considerable attention, since antagonistic yeasts have simple nutritional requirements and many of them have been proven to be harmless to potential consumers (Zhimo et al., 2014; Sui et al., 2015; Ocampo-Suarez et al., 2017). A growing body of new antagonists has been isolated from environment, and many biocontrol products have been developed and are mostly available in the agricultural markets in North America and Europe (Borriss, 2015). However, several of these antagonists have not achieved commercial success due to inconsistent results, and most of the biocontrol products have limited application on fruit crops (Droby et al., 2016). In contrast, *Metschnikowia fructicola* (Shemer, Bayer, Leverkusen, Germany) is a more successful biocontrol product for pre- and post-harvest applications on various fruits and vegetables, which registered in Israel and acquired by Bayer CropScience (Germany) and then delegated to Koppert (Netherlands) (Droby et al., 2016). In the genus *Metschnikowia*, not only *M. fructicola*, *Metschnikowia pulcherrima* and *Metschnikowia andauensis* have also been used as postharvest biocontrol agents (Kurtzman and Droby, 2001; Spadaro and Gullino, 2004; Manso and Nunes, 2011). *Metschnikowia citriensis,* an effective biocontrol agent for citrus postharvest green and blue molds, was identified in our previous study (Liu et al., 2017, 2018b, 2019). Iron depletion by forming maroon pigment has been known as an important action mechanism of *Metschnikowia* against *Botrytis cinerea*, *Alternaria alternata*, *Penicillium digitatum*, *Penicillium expansum*, *Penicillium italicum*, and *Aspergillus oryzae* (Saravanakumar et al., 2008; Türkel et al., 2014; Liu et al., 2019). Ferric ions and pulcherriminic acid non-enzymatically form pulcherrimin that is the maroon pigment produced by *Metschnikowia* (MacDonald, 1965; Sipiczki, 2006). In addition to iron depletion, the strong ability of *M*. *citriensis* to adhere tightly to the mycelia of pathogens and the surface of fruit as well as form biofilm was also hypothesized to play a key role in its biocontrol activity (Liu et al., 2019). Biofilms are spatially organized and dense communities of microorganisms, and the extracellular polymeric substances (EPS) produced by them consist mainly of water and extracellular biopolymers (polysaccharides, proteins, DNA, and lipids), making them have strong adhesion (Pandin et al., 2017). Biocontrol agent biofilms on the surface of the host tissue that prevent new growth of pathogens and avoid their invasion greatly improve the biocontrol efficacy of antagonistic yeasts against postharvest diseases (Liu et al., 2013).

Biocontrol yeasts do not show the same effect comparable to synthetic fungicides, because the biotic and abiotic stresses such as oxidative, pH, temperature, UV, and osmotic stresses impact the yeast viability and biocontrol activity (Liu et al., 2011, 2018a; Zhao et al., 2012; Sui et al., 2015). When yeast cells are subjected to these environmental stresses, large amounts of ROS accumulate in yeast cells, resulting in severe oxidative damage (Liu et al., 2013; Sui et al., 2015). There is some evidence that yeast antagonists produce relatively large amounts of superoxide anions ($O_{2}^{•-}$) and induce transient ROS production in the host (Macarisin et al., 2010). In addition, H_2_O_2_ accumulation in response to wounding and pathogen attack in apple and citrus fruit has been studied (Torres et al., 2003, 2011; Macarisin et al., 2007; Su et al., 2011; Buron-Moles et al., 2015). These ROS play a signaling role in the host by controlling the redox of transcription factors or by interacting with other signaling components (e.g., the mitogen-activated protein kinase cascade) to mediate defense gene activation (Zhang and Klessig, 2001; Macarisin et al., 2010; Hershkovitz et al., 2012). The oxidative burst in fruit wounds is due to the reactive oxygen species produced by the yeast and the host, and large amounts of H_2_O_2_ are detected in the yeast cells collected from fruit wounds (Droby et al., 2009; Piombo et al., 2018). Survival and reproduction in host wounds are essential for postharvest biocontrol yeasts, and thus finding effective approaches to improve their oxidative stress tolerance is necessary to improve their biocontrol ability.

Proline has showed an antioxidant feature, suggesting direct ROS scavenging feature, functioning as a molecular chaperone able to enhance the activities of ROS scavenging enzymes, and activating alternative detoxification pathways (Matysik et al., 2002; Hoque et al., 2008; Szabados and Savoure, 2010). Proline treatment can diminish ROS levels in fungi and thus inhibits ROS-induced apoptosis (Chen and Dickman, 2005). Under oxidative stress, apoptosis has been reported as a major cause of loss of cell viability for biocontrol yeasts (Chen et al., 2015; Zhang et al., 2017). In addition to its antioxidant feature, proline can also stabilize cellular homeostasis during stress through proline metabolism (Szabados and Savoure, 2010). Although several studies have reported on the strategies to improve oxidative stress resistance of antagonistic yeasts, the application of proline is remain rare. In addition, our preliminary unreported data showed that proline could induce the pigment production of *M*. *citriensis* that plays an important role in inhibition of pathogens by depleting iron, but we know little about its role in response to oxidative stress.

The aim of this study was to investigate the effect of proline on oxidative stress tolerance and biocontrol ability of *M*. *citriensis*, and the possible mechanisms involved. Moreover, the role of pigment production was elucidated. More specifically, we investigated (1) the survival of *M*. *citriensis* exposed to oxidative stress stimulated by different concentrations of H_2_O_2_; (2) the effect of proline on oxidative stress tolerance, ROS accumulation, membrane integrity, and apoptosis of *M*. *citriensis* cells; (3) the effect of proline on antioxidant enzymes, including CAT and SOD in *M*. *citriensis*; and (4) the effect of proline on population dynamics, biofilm formation, and biocontrol ability of *M*. *citriensis* against *P. digitatum* on citrus fruits.

## Materials and Methods

### Yeast and Pathogen

*M. citriensis* strain FL01^T^, an epiphytic yeast of citrus leaves (Liu et al., 2018b), was stored in a tube with nutrient yeast dextrose agar (NYDA) medium [5 g/L yeast extract, 8 g/L nutrient broth, 10 g/L dextrose, 20 g/L agar (Aobox, China)]. The yeast culture was incubated at 28°C at 200 rpm for 16 h to reach the mid-log phase from an initial concentration of 1 × 10^5^ cells/ml. In addition, the *P. digitatum* strain was obtained from citrus fruits showing symptoms of disease, and its internal transcribed spacer (ITS) region was sequenced (unpublished data). The mold stock culture was incubated at 25°C for 7 days on potato dextrose agar (PDA) medium [200 g/L potatoes, 20 g/L dextrose, 20 g/L agar (Aobox, China)], and spores suspension was obtained by flooding the spores of the 7-day culture with sterile distilled water (SDW) and then filtering through four layers of sterile gauze.

### Fruits

Citrus fruits [*Citrus sinensis* (L.) Osbeck cv. Olinda Valencia orange] were harvested at commercial harvest maturity from a conventional orchard (Zhongxian, Chongqing). The fruits were superficially disinfected with sodium hypochlorite (2% v/v) for 2 min, washed with tap water, and dried in the air prior to further use.

### Evaluation of Survival of *M*. *citriensis* Under Oxidative Stress

The oxidative stress tolerance of *M*. *citriensis* was investigated according to the method of Zhang et al. (2017) with slight modification. The mid-log phase yeast cells were harvested by centrifugation (8,000 × *g*, 5 min) and then washed twice with SDW. The yeast cells were re-suspended in fresh nutrient yeast dextrose broth (NYDB), and the concentration was adjusted to 5 × 10^7^ cells/ml and then exposed to 0, 20, 40, 60, and 80 mM H_2_O_2_ at 28°C, 150 rpm for 90 min. After that, yeast cells were collected by centrifugation and then washed twice. Serial 10-fold dilutions of the samples to 5 × 10^3^ cells/ml were made, and 50 μl of diluted cultures was spread on NYDA medium. After 2 days of incubation at 28°C, the number of colony-forming units per milliliter (CFU/ml) was calculated. The viability of *M. citriensis* under moderately lethal oxidative stress was expressed as a percentage of the number of colonies with and without H_2_O_2_ exposure. For each treatment, there were three replicates and the experiment was repeated three times.

### Tests for Pigment Production and Intracellular Iron Content

A loopful of the cell suspension (1 × 10^8^ cells/ml) of *M. citriensis* was streaked onto the NYDA plate amended with or without 1 mM proline to test pigment production. Intracellular iron levels were measured according to the method of Xu et al. (2014) with slight modification, relying on the phenomenon that bathophenanthroline disulfonate (BPS) forms a colored complex with iron. The yeast cells (1 × 10^5^ cells/ml) were incubated in NYDB medium (control) and NYDB medium supplemented with (1) 1 mM proline (the determination of concentration was based on preliminary unreported data), (2) 10 μM tropolone that has strong chelating ability for iron ions, and (3) 10 μM FeCl_3_. The mid-log phase yeast were harvested by centrifugation and washed twice with SDW and then re-suspended in 500 μl HNO_3_ (3%). When the cells are completely digested after boiled for 2 h, 400 μl of supernatant was transferred to a new tube and mixed with 160 μl ascorbic acid (34 mg/ml), 126 μl ammonium acetate (4 M), and 320 μl BPS (1.7 mg/ml). After incubation for 10 min at room temperature, the absorbance of the BPS-Fe complex at 535 nm and the nonspecific absorbance at 680 nm were measured by the Multiskan Spectrum microplate spectrophotometer (BioTek Instrument Inc., USA). The iron content was displayed in arbitrary units (A.U.) and calculated as follows: (OD_535_ − OD_680_)/the number of cells. For each treatment, there were three replicates and the experiment was repeated three times.

### Effect of Proline on Oxidative Stress Tolerance of *M*. *citriensis*

The effect of proline on cell viability of *M*. *citriensis* after exposure to H_2_O_2_ was determined according to the method of Zhang et al. (2017), with some modification. The yeast cells were incubated in NYDB medium and NYDB medium containing (1) 1 mM proline and (2) 10 μM tropolone, and the cell concentration was adjusted to 1 × 10^5^ cells/ml. After overnight cultivation, cells were harvested by centrifugation. The cells were washed three times with SDW and then re-suspended in fresh NYDB medium, and the concentration was adjusted to 5 × 10^7^ cells/ml. After that, the cells were exposed to 40 mM H_2_O_2_ for 90 min. Untreated yeast cells without exposure to H_2_O_2_ were used as controls. Survival rates were evaluated by the aforementioned method. Moreover, after exposure to oxidative stress, the concentration of yeast cells was adjusted to 1 × 10^6^ cells/ml for the yeast spotting assay, and then a 5 μl yeast sample was seeded onto NYDA medium and cultured at 28°C. For each treatment, there were three replicates and the experiment was repeated three times.

### Imaging of Intracellular ROS

The intracellular ROS production in *M*. *citriensis* was detected according to the method of Liu et al. (2011) with minor modification, using 2′, 7′-dichlorodihydrofluorescein diacetate (DCHF-DA, Molecular Probe) (Sigma, USA), a oxidant-sensitive probe. The mid-log phase yeast cells (untreated, proline-treated, tropolone-treated, and FeCl_3_-treated) were harvested as described above and then treated with 40 mM H_2_O_2_ for 90 min. After that, yeast cells were harvested by centrifugation and then washed twice with phosphate buffered saline (PBS, pH 7.0). The yeast cells were re-suspended with an equal volume of PBS with DCHF-DA at a final concentration of 25 μM and then incubated at 30°C in the dark. After an hour, yeast cells were washed twice with PBS and then detected under an Eclipse TS100 epifluorescence microscope (Nikon Instrument Inc., Japan) with excitation at 485 nm and emission at 530 nm. The percentage of stained cells was calculated, and each slide was randomly selected for six fields of view, each field containing at least 100 cells. Untreated yeast cells without exposure to H_2_O_2_ were used as controls. For each treatment, there were three replicates and the experiment was repeated three times.

### Analysis of Membrane Integrity and Apoptosis of *M*. *citriensis* Cells Under Oxidative Stress

Membrane integrity and apoptosis of *M*. *citriensis* cells were analyzed according to the method of Chen et al. (2015), with minor modification. The mid-log phase yeast cells (untreated, proline-treated, tropolone-treated, and FeCl_3_-treated) were harvested as described above and then treated with 40 mM H_2_O_2_ for 90 min. Untreated yeast cells without exposure to H_2_O_2_ were used as controls. After that, yeast cells were harvested by centrifugation and then washed twice with PBS (pH 7.0). The yeast cells were re-suspended with an equal volume of PBS containing propidium iodide (PI, 10 μg/ml) and Hoechst 33342 (5 μg/ml) for 20 min at room temperature in the dark and then washed twice with PBS. After that, yeast cells were observed using an Eclipse TS100 epifluorescence microscope. PI-positive yeast cells indicated dead cells with damaged plasma membranes, including necrosis, necroptosis, and pyroptosis (Dixon and Stockwell, 2014; Nagata and Nakano, 2017). Hoechst 33342-positive and PI-negative cells indicated apoptotic cells (Hong et al., 2007). The percentage of apoptotic cells and plasma membrane integrity were calculated respectively, and each slide was randomly selected for six fields of view, each field containing at least 100 cells. For each treatment, there were three replicates and the experiment was repeated three times.

### Assays of CAT and SOD Activities

Yeast cells (untreated and proline-treated) at mid-log phase were harvested by centrifugation as described above and then exposed to 40 mM H_2_O_2_. Extracts of enzymes of CAT and SOD were prepared according to the method of Zhang et al. (2017), with some modification. The yeast cells were collected by centrifugation (8,000 × *g*, 5 min) at specific intervals (0, 30, 60, and 90 min) after being treated with 40 mM H_2_O_2_, and then washed twice with SDW. The cells were suspended in cold 50 mM PBS (pH 7.0) containing 2 mM phenylmethanesulfonyl fluoride and 1 mM EDTA for CAT and SOD extraction by vortexing with glass beads to break the cell walls. After centrifugation at 10,000 × *g* for 20 min at 4°C, the supernatant was enzyme extract.

One hundred microliters of enzyme extract was mixed with 1.4 ml of 40 mM H_2_O_2_ to test CAT activity, and the absorbance at 240 nm was measured every 30 s for 5 min to determine the decomposition of H_2_O_2_. Inactivated enzyme extracts boiled for 5 min were used as controls. One unit of CAT activity was the amount of enzyme required to decompose 1 μM H_2_O_2_ per min. The reaction mixtures with SOD consisted of 50 μl enzyme extract and 2.95 ml 50 mM PBS containing 2 μM riboflavin, 13 mM methionine, 10 μM EDTA, and 75 μM nitroblue tetrazolium. After being illuminated by light (4,000 lx) for 20 min, the absorbance at 560 nm was determined. Non-illuminated solutions were used as controls. One unit of SOD activity was the amount of enzyme, which causes 50% of NBT reduction. The activities of both enzymes were expressed as U/mg protein. The determination of protein was based on the method of Bradford (1976). For each treatment, there were three replicates and the experiment was repeated three times.

### Population Growth of *M*. *citriensis* in Wounds

The mid-log phase yeast cells (untreated and proline-treated) were harvested and then treated with 40 mM H_2_O_2_ for 90 min as described above. Untreated yeast cells without exposure to H_2_O_2_ were used as a control. The wounds of fruit samples (approximately 3 mm wide × 2 mm deep) were made with a sterile needle on superficially sanitized citrus fruits (three wounds per fruit). Twenty microliters of yeast suspension (1 × 10^8^ cells/ml) was individually pipetted to the wounds, and then fruits were placed in plastic bags either incubated at 25 or 4°C. Tissue samples containing the whole wound of fruits were extracted after 0, 2, 4, 6, and 8 days of incubation at 25°C (after a 1-h incubation as the time 0), and the samples of fruits were extracted after 0, 5, 10, 15, and 20 days of incubation at 4°C, using a sterile cork borer. Each sample was ground in 10 ml PBS using a pestle and mortar, and 50 μl of diluted cultures was spread on NYDA plates after making serial 10-fold dilutions. Colonies were counted after 48 h of incubation at 28°C, and the population density was expressed as the Log_10_ CFU/wound. For each treatment, there were three replicates with five fruits per replicate and the experiment was repeated twice.

### Biocontrol Assay of *M*. *citriensis*

Biocontrol ability of *M*. *citriensis* was evaluated on citrus fruits according to the method of Liu et al. (2017), with slight modification. The untreated (Treatment II) and proline-treated (Treatment III) yeast cells were harvested as described above and then treated with 40 mM H_2_O_2_ for 90 min. Untreated yeast cells without exposure to H_2_O_2_ were cells of Treatment I. Two wounds of fruit samples (approximately 3 mm wide × 2 mm deep) were made at the equator, using a sterile needle. Twenty microliters of yeast suspension (1 × 10^8^ cells/ml) of each treatment group was inoculated into each wound, and the wounds inoculated with SDW were used as controls. After 4 h, 10 μl of *P. digitatum* spores suspension (1 × 10^5^ spores/ml) was pipetted to each wound. The treated fruits were then stored in enclosed plastic trays at 25 and 4°C, putting wet paper towels in the trays to increase the humidity. The disease incidence (DI) and lesion diameter (ID) of the fruits were recorded daily and every 3 days, respectively, after ID of the controls grew to measurable sizes. The DI of treated fruits was calculated as a percentage of the number of decayed wounds and total wounds, and the LD was computed as mean value of long and short diameters of the damaged area. Fruits with LD more than 3mm (the size of wound) were considered to be decayed. For each treatment, there were three replicates with five fruits per replicate and the experiment was repeated twice.

### Biofilm Formation of *M*. *citriensis*

Biofilm formation ability of *M*. *citriensis* on citrus fruits was analyzed using scanning electron microscopy (SEM). The fruits were treated and maintained as described above. After incubation for 26 days at 4°C, tissue samples were taken with a sterile knife and washed three times with 0.1 mM PBS (pH 6.8). Samples were then fixed in 0.1 mM PBS containing 2.5% (v/v) glutaraldehyde for 12–24 h at 4°C and dehydrated in ethanol series (30, 50, 70, 80, 90, and 100% twice). After that, the ethanol was replaced by tertiary butyl alcohol series (50, 70, 90, 95, and 100% twice). The samples were then dried at 65°C for 2 h with a DZF-6051 vacuum drying oven (Shanghai Jinghong Experimental Equipment Co., Ltd., China), and the dried samples were coated with a thin layer of gold before viewing in a JEOL JSM-6510LV SEM (JEOL Ltd., Japan).

Biofilm formation ability of *M*. *citriensis in vitro* was investigated according to the method of Parafati et al. (2015) with slight modification, by measuring the cell adherence to the surface of a polystyrene plate. The yeast cells with an initial concentration of 1 × 10^5^ cells/ml were incubated in NYDB medium amended with or without 1 mM proline and YNB medium supplemented with different concentrations of iron (0, 1, 10, 100 μM). After overnight cultivation, the cells were harvested and washed twice with PBS (pH 7.2). The cell density was adjusted to 1 × 10^7^ cells/ml with yeast nitrogen base (YNB) containing 100 mM glucose, and 100 μl of the cell culture was inoculated into wells of a 96-well polystyrene plate. The wells without yeast suspensions served as controls. After incubation for specific intervals (0, 30, 60, 120 min) at 28°C on a rotary shaker at 75 rpm, the wells were washed twice with PBS. One hundred microliters of 0.4% crystal violet solution was then added to the wells. After being stained for 45 min, the wells were washed four times with SDW. Subsequently, the wells were destained with 200 μl of 95% ethanol for 45 min, and then 100 μl of this solution was transferred to a new 96-well plate. The biofilm formation ability was expressed as crystal violet content and calculated as follows: OD_590_ of the test wells were subtracted that of the controls. For each treatment, there were three replicates and the experiment was performed twice.

Mutagenesis and mutant characterization were according to the method of Sipiczki (2006), with slight modification. Yeast cells in overnight NYDB culture (28°C, 100 rpm for 12 h) were collected by centrifugation (8,000 × *g*, 5 min, 10°C). The cells were re-suspended in fresh NYDB medium containing 300 μg/ml 1-methyl-3-nitro-1-nitrosoguanidine (Sigma-Aldrich, USA), and the cell concentration was adjusted to 1 × 10^7^ cells/ml and then incubated at 28°C, 100 rpm for 30 min. After that, diluted cultures were spread on PDA plates supplemented with 10 μg/ml FeCl_3_ and incubated at 28°C for 3 days without light. The colonies that have narrower pigmented halos and are lighter in color than wild-type *M. citriensis* were isolated, and the mutagenized isolates whose growth ability that evaluated by OD_600_ significantly weaker than that of wild-type were discarded. The remaining candidate mutagenized isolates were then purified by single cell separation. To test the stability of their low-pigment phenotype, the mutagenized isolates were cultured at least 5 times. A loopful of the cell suspension (1 × 10^8^ cells/ml) was streaked onto the PDA plates to test pigmentation, and the biofilm formation *in vitro* of the mutants was tested as described above.

### Statistical Analysis

Data from experiments were analyzed by using SPSS 22.0 (SPSS Inc., Chicago, IL, USA). The arithmetic means were calculated and analyzed by using one-way analysis of variance (ANOVA) in all repeated experiments. The difference between the means were analyzed by Duncan’s multiple range test and considered significant at *p* < 0.05.

## Results

### Evaluation of Survival of *M*. *citriensis* Under Oxidative Stress

**Figure 1:**
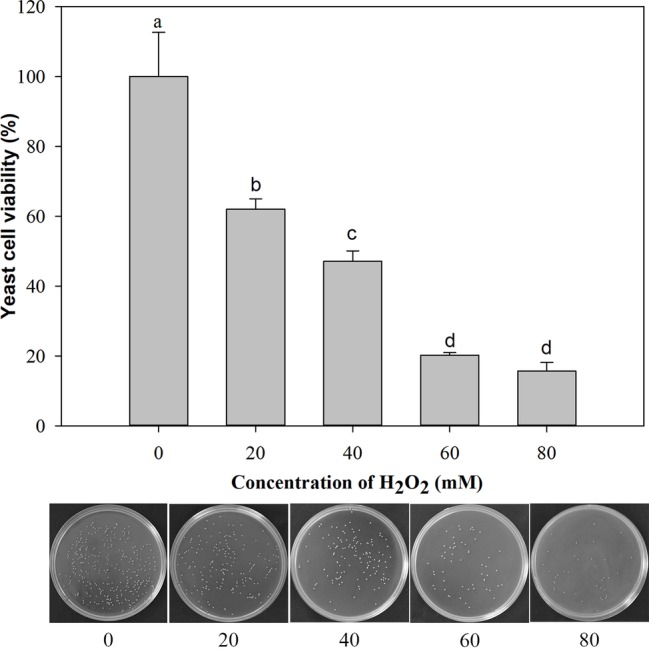
Viability of *Metschnikowia citriensis* exposed to oxidative stress (a series of concentrations of H_2_O_2_) for 90 min. The image of petri dishes shows the number of colony-forming units on NYDA medium after treatment with a series of concentrations of H_2_O_2_. Vertical bars represent standard errors of the mean. Data in columns with different letters are significantly different according to Duncan’s multiple range test (*p* < 0.05).

Under the oxidative stress of H_2_O_2_ (ranging from 0 to 80 mM) for 90 min, the cell viability decreased gradually with the increasing concentration of H_2_O_2_. The cells exposed to 20 mM H_2_O_2_ exhibited 62% viability and 47% at 40 mM H_2_O_2_. Therefore, the oxidative stress imposed by 40 mM H_2_O_2_ for 90 min was semi-lethal to *M*. *citriensis* cells, and this oxidative stress condition was chosen for subsequent experiments (Figure 1).

### Tests for Pigment Production and Intracellular Iron Content

**Figure 2:**
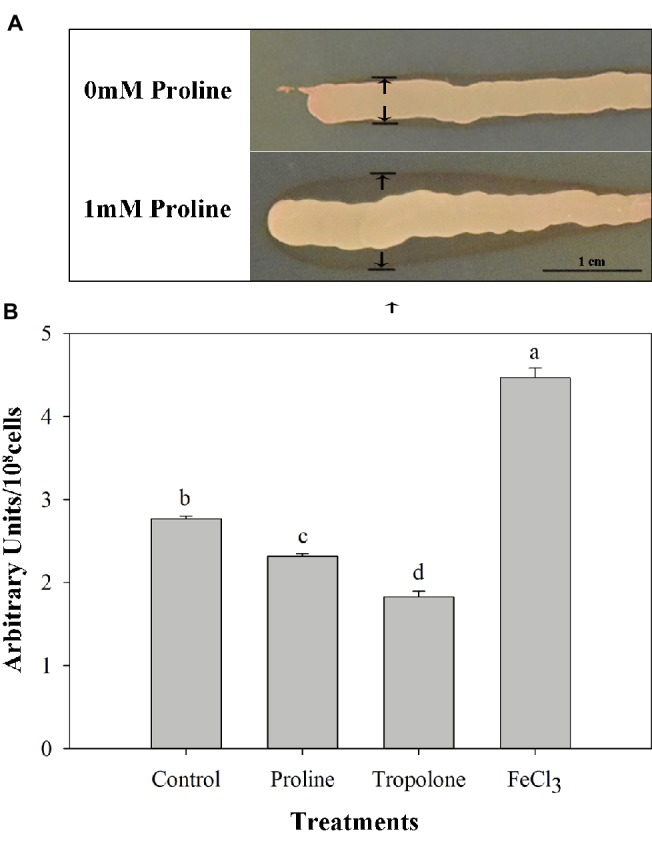
The effect of proline treatment on pigment production and intracellular iron content of *Metschnikowia citriensis*. **(A)** Pigment production of *M*. *citriensis* on NYDA plates amended with or without proline. **(B)** Metal iron contents in the untreated (control) and proline-treated cells were quantified by the BPS-based colorimetric method. The tropolone-treated cells were used as a positive control, and FeCl_3_-treated cells were used as a negative control. Vertical bars represent standard errors of the mean. Data in columns with different letters are significantly different according to Duncan’s multiple range test at a 5% level.

Proline treatment increased the pigment production of *M*. *citriensis* (Figure 2A), since proline-treated cells showed wider pigmented halo on NYDA plates compared with untreated yeast cells (control). In addition, the intracellular iron content of *M*. *citriensis* incubated in the NYDB medium containing proline or iron chelator tropolone was shown to be significantly lower, while that of FeCl_3_-treated cells were significantly higher than the control (Figure 2B). Therefore, maroon pigment formed by chelated iron decreased the intracellular iron content of *M*. *citriensis*.

### Effect of Proline on Oxidative Stress Tolerance of *M*. *citriensis*

**Figure 3:**
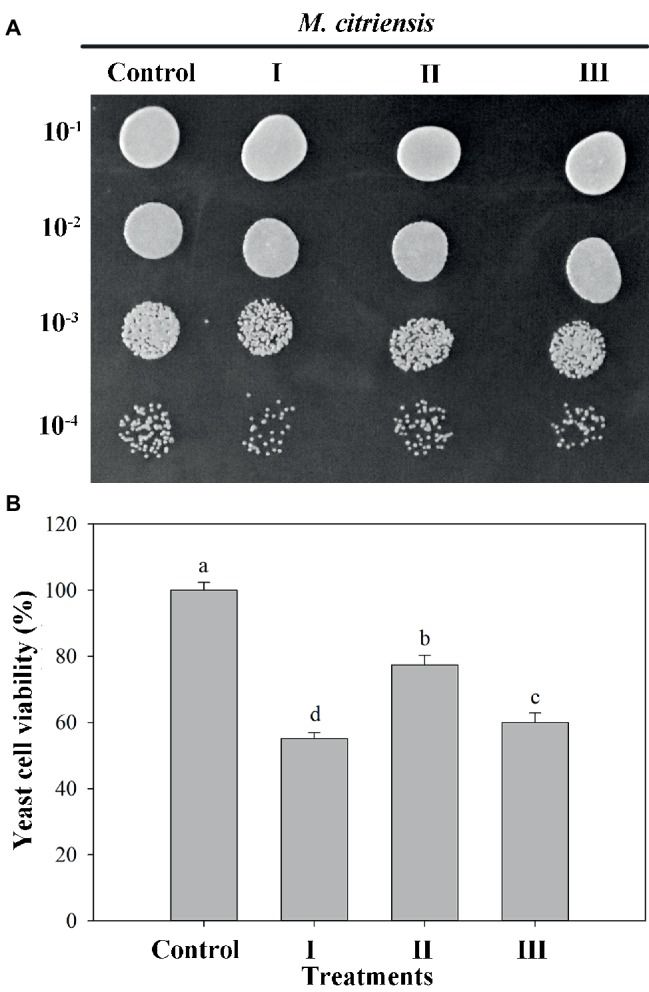
Viability of *Metschnikowia citriensis* under moderately lethal oxidative stress. **(A)** Image of the yeast spotting assay of viability of *M*. *citriensis* following exposure to 40 mM H_2_O_2_. **(B)** Viability of yeast cells following exposure to 40 mM H_2_O_2_. (I) Cells harvested from NYDB medium, (II) cells harvested from NYDB medium containing 1 mM proline, and (III) cells harvested from NYDB medium containing 10 μM tropolone were exposed to 40 mM H_2_O_2_ for 90 min. The cells harvested from NYDB medium without exposure to H_2_O_2_ were used as controls. The vertical bars represent standard errors of three replicates. Data in columns with different letters are significantly different according to the Duncan’s multiple range test (*p* < 0.05).

The viability of both proline-treated and untreated (control) yeast cells exposed to 40 mM H_2_O_2_ for 90 min was measured. Oxidative stress imposed by H_2_O_2_ significantly reduced the survival rate of *M*. *citriensis*. As indicated in Figure 3, the viability of *M*. *citriensis* could be greatly increased by proline (77%), compared with the control. Though the cell viability of *M*. *citriensis* incubated in NYDB amended with tropolone (60%) was not as high as that incubated in the NYDB amended with proline, tropolone-treated cells also showed a modest increase in cell viability compared with the control (55%).

### Imaging of Intracellular ROS

**Figure 4:**
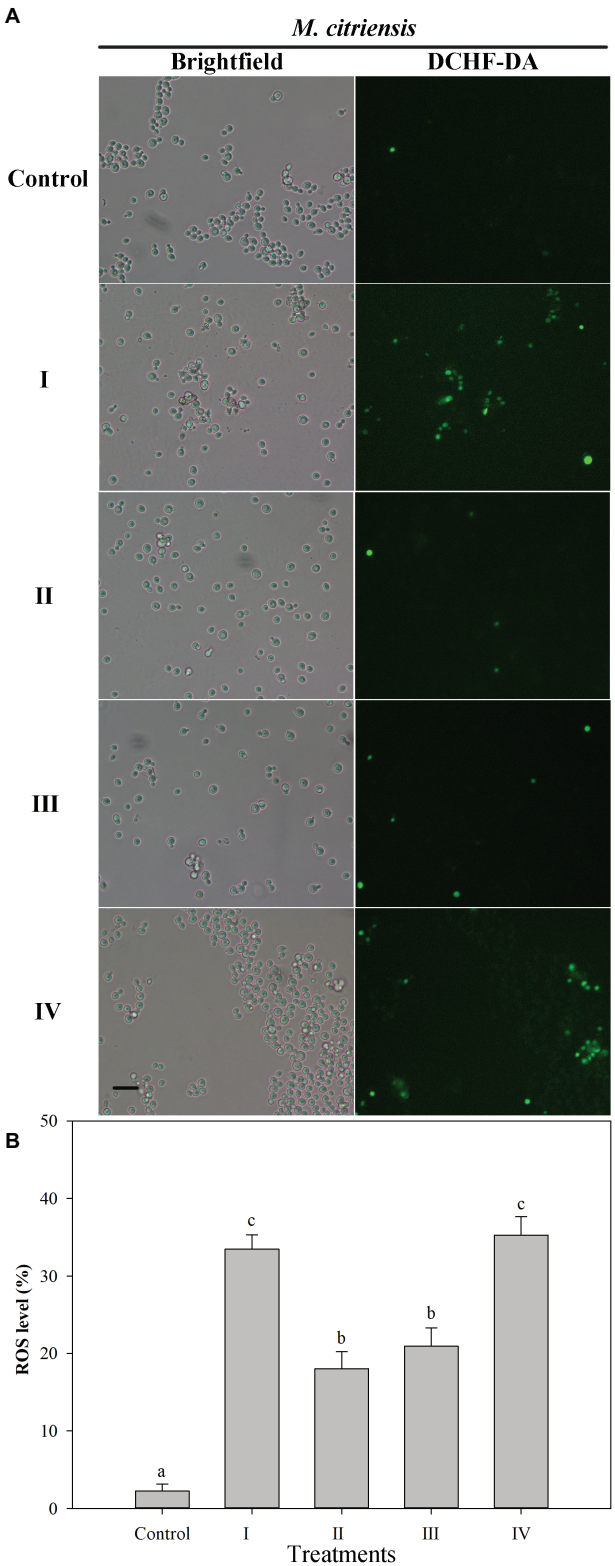
Intracellular ROS level in *Metschnikowia citriensis* under oxidative stress. **(A)** Fluorescence microscopic images of *M*. *citriensis* cells stained with the fluoroprobe DCHF-DA. **(B)** Percentage of *M*. *citriensis* cells exhibiting visible ROS accumulation. (I) Cells harvested from NYDB medium, (II) cells harvested from NYDB medium containing 1 mM proline, (III) cells harvested from NYDB medium containing 10 μM tropolone, and (IV) cells harvested from NYDB medium containing 10 μM FeCl_3_ were exposed to 40 mM H_2_O_2_ for 90 min. The cells harvested from NYDB medium without exposure to H_2_O_2_ were used as controls. The vertical bars represent standard errors of three replicates. Data in columns with different letters are significantly different according to the Duncan’s multiple range test (*p* < 0.05). Bar = 20 μm.

Intracellular ROS production was determined using the fluorescent dye DCHF-DA (Figure 4). Prior to H_2_O_2_ treatment (control), the percentage of ROS-positive cells was 2.3%. However, after H_2_O_2_ treatment for 90 min, the percentages of ROS of proline-treated and untreated cells exhibiting a visible ROS level were 18.0 and 33.5%, respectively. These results showed that oxidative stress could cause large amount accumulation of intracellular ROS in *M*. *citriensis*, but proline treatment decreased intracellular ROS level. In addition, tropolone-treated cells showed a significantly lower ROS level, compared with untreated cells.

### Analysis of Membrane Integrity and Apoptosis of *M*. *citriensis* Under Oxidative Stress

**Figure 5:**
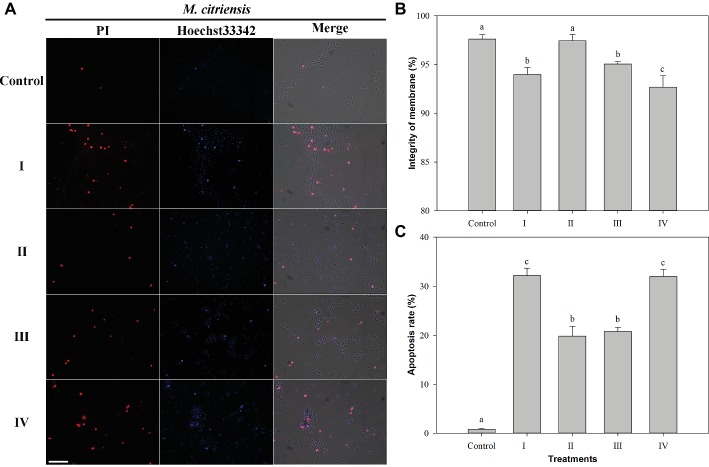
Membrane integrity and apoptosis analysis of *Metschnikowia citriensis* under oxidative stress. **(A)** Fluorescence microscopic images of *M*. *citriensis* cells double stained with PI/Hoechst 33342. **(B)** Percentage of plasma membrane integrity of *M*. *citriensis* cells. **(C)** Apoptosis rate of *M*. *citriensis* cells. (I) Cells harvested from NYDB medium, (II) cells harvested from NYDB medium containing 1 mM proline, (III) cells harvested from NYDB medium containing 10 μM tropolone, and (IV) cells harvested from NYDB medium containing 10 μM FeCl_3_ were exposed to 40 mM H_2_O_2_ for 90 min. The cells harvested from NYDB medium without exposure to H_2_O_2_ were used as controls. The vertical bars represent standard errors of three replicates. Data in columns with different letters within each parameter are significantly different according to the Duncan’s multiple range test (*p* < 0.05). Bar = 20 μm.

Membrane integrity and apoptosis of *M. citriensis* were demonstrated using Hoechst 33342 and PI staining (Figure 5). The dead yeast cells with damage of plasma membranes were detectable, since they were stained by PI and hence appeared red fluorescence (Figure 5A). Following exposure to 40 mM H_2_O_2_ for 90 min, the PI-negative/Hoechst33342-positive apoptotic cells (Figure 5C) and the dead yeast cells with damaged plasma membranes with PI-positive/Hoechst33342-positive (Figure 5B) increased to 32 and 6%, respectively. Proline treatment decreased the apoptosis to 20% and increased the plasma membrane integrity (97%) to the level comparable to untreated cells without exposure to H_2_O_2_ (control). Similarly, tropolone treatment also decreased the apoptosis to 21%. In addition, the dead yeast cells with damaged plasma membranes (7%) increased through cultivation in the NYDB media amended with FeCl_3_. These results indicated that exposure of *M. citriensis* cells to exogenous H_2_O_2_ can trigger iron-dependent apoptosis, which can be inhibited by an iron chelator, and increased iron concentration can promote plasma membrane damage under oxidative stress.

### Assays of CAT and SOD Activities

**Figure 6:**
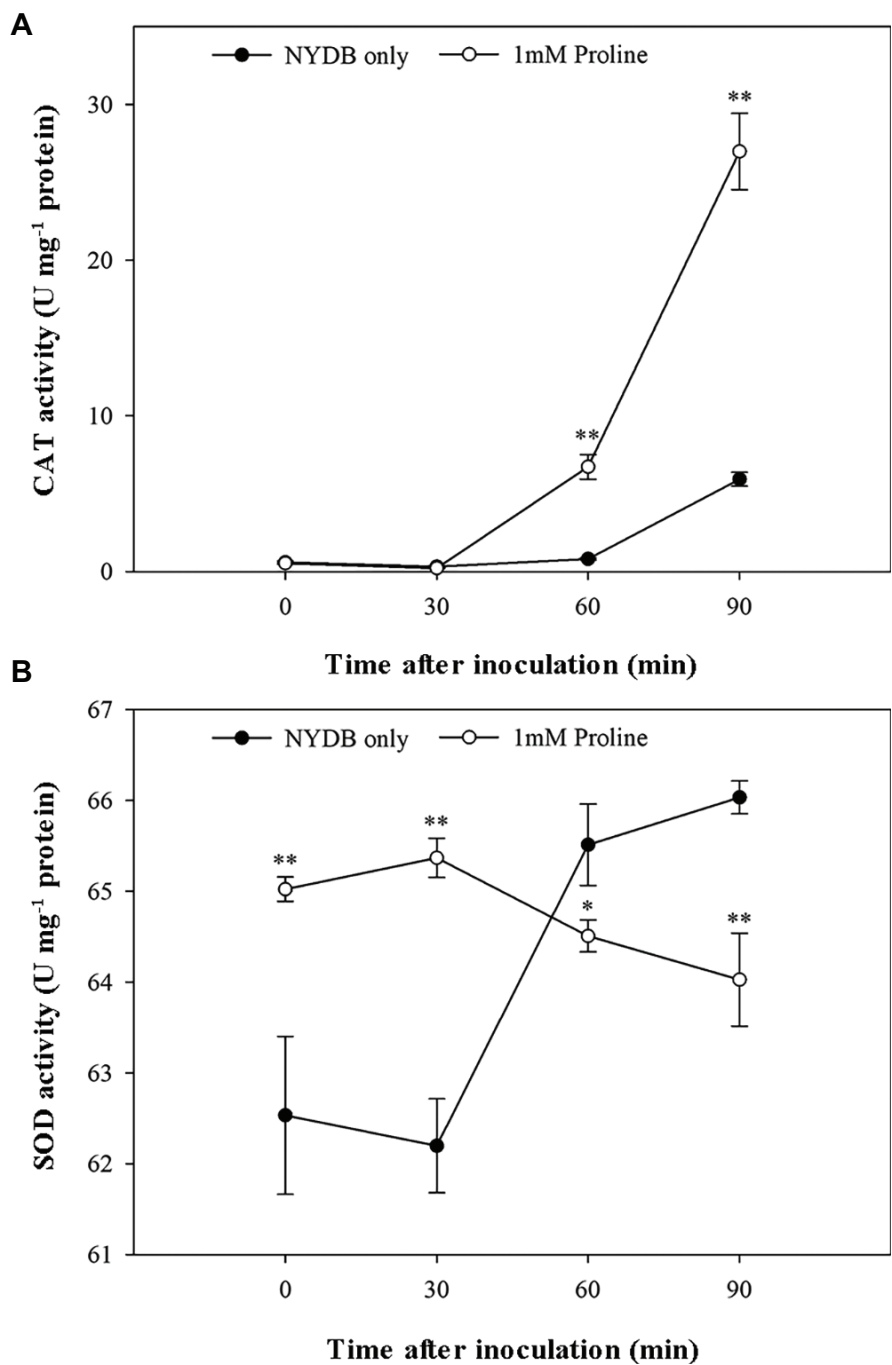
CAT and SOD activity of *Metschnikowia citriensis* following treatment with moderately lethal concentrations of H_2_O_2_. **(A)** CAT activity of *M*. *citriensis* harvested from NYDB medium with or without proline. **(B)** SOD activity of *M*. *citriensis* harvested from NYDB medium with or without proline. The vertical bars represent standard errors of three replicates. “*” indicates a significant difference according to the Duncan’s multiple range test (*p < 0.05, **p < 0.01).

Given that antioxidants, including CAT and SOD, are critical to protecting cells against oxidative stress by maintaining $O_{2}^{•-}$ and H_2_O_2_ at low levels (Chen and Dickman, 2005), the status of the activities of these scavenging enzymes in *M. citriensis* under moderately lethal oxidative stress was evaluated. Following exposure to 40 mM H_2_O_2_ for 30 min, the untreated cells only showed a slight increase in CAT activity. Interestingly, proline-treated cells caused a nearly eightfold increase in CAT activity compared with untreated cells after 60 min of exposure to H_2_O_2_, and CAT activity continued to rise up to 90 min (Figure 6A). Moreover, addition of proline to the medium of *M. citriensis* increased SOD activity (time 0), and SOD activity of the proline-treated cells maintained a higher level than that of the untreated cells after 0–30 min of exposure to H_2_O_2_ (Figure 6B). In contrast, exogenous oxidative stress did not increase SOD activity of the untreated cells until 30 min of incubation while that in the proline-treated cells started to decrease. These data suggested that proline treatment induced a significant increase in SOD activity of *M. citriensis* in the early stage and increased CAT activity in the later stage, during oxidative stress.

### Population Growth of *M. citriensis* in Wounds

**Figure 7:**
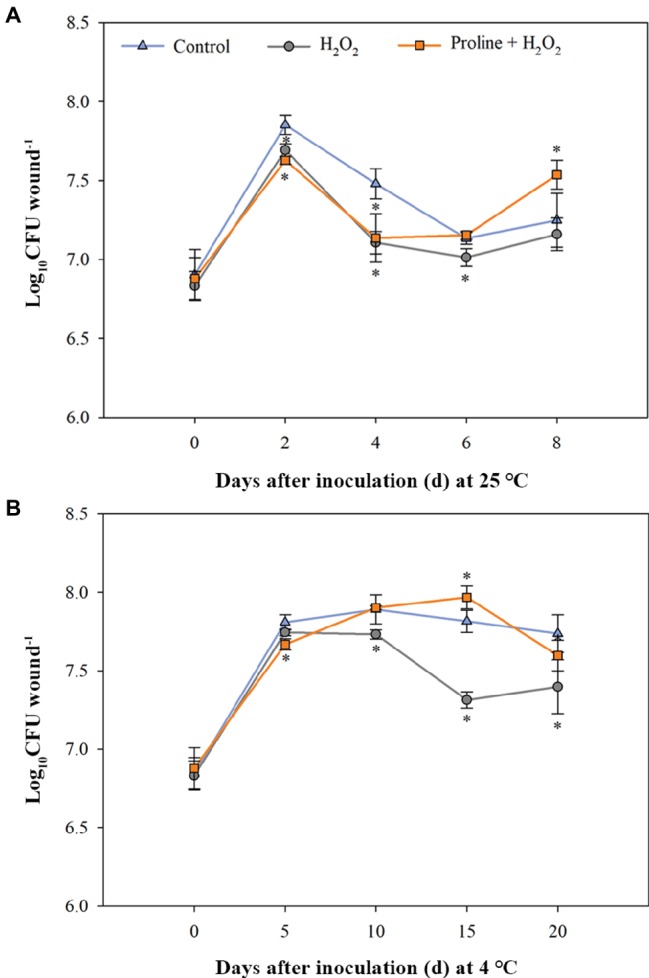
Population dynamics of *Metschnikowia citriensis* in wounds of citrus fruits stored at 25°C **(A)** and 4°C **(B)**. The *M*. *citriensis* cells harvested from NYDB medium with or without 1 mM proline were exposed to 40 mM H_2_O_2_ for 90 min and then seeded to the wounds. The cells harvested from NYDB medium without exposure to H_2_O_2_ were used as controls. The vertical bars represent standard errors of three replicates. “*” indicates a significant difference from the control according to the Duncan’s multiple range test (*p* < 0.05).

The population growth of *M*. *citriensis* harvested from citrus wounds incubated at 25 and 4°C is shown in Figure 7. The untreated cells without exposure to H_2_O_2_ (control) multiplied quickly in the wounds and reached a higher level of population density at 25°C (log_10_ CFU/wound = 7.9). Moreover, the population of control cells reached a maximum (log_10_ CFU/ wound = 7.9) earlier than the untreated cells with exposure to H_2_O_2_ at 4°C. The untreated cells with exposure to H_2_O_2_ showed significantly lower populations than the control at almost all time points at 25 and 4°C (Figures 7A,B). These results indicated exogenous oxidative stress significantly inhibited the growth of *M. citriensis* cells. However, proline increased the population density of *M. citriensis* in wounds late in the storage (Figures 7A,B). After 8 days of incubation at 25°C, *M*. *citriensis* treated with 1 mM proline and 40 mM H_2_O_2_ showed a marked higher population than the untreated cells with exposure to H_2_O_2_ and the control (Figure 7A). Moreover, the population density of proline-treated cells with exposure to H_2_O_2_ reached a higher level at 4°C (log_10_ CFU/wound = 8.0) than the untreated cells with exposure to H_2_O_2_ and the control, though the cell growth rate was not as quick as that of the control (Figure 7B).

### Biocontrol Assay of *M. citriensis*

**Figure 8:**
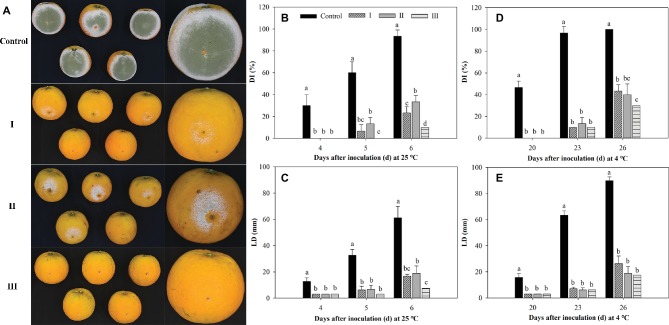
Effect of oxidative stress and proline treatment on the efficacy of *Metschnikowia citriensis* against *Penicillium digitatum* on citrus fruits. **(A)** Biocontrol performance of *M*. *citriensis* cells against *P. digitatum* on the 5th day after inoculation at 25°C. Statistical analysis of **(B)** decay incidence (DI) and **(C)** lesion diameters (LD) on citrus fruits after 4, 5, and 6 days of inoculation at 25°C. Statistical analysis of **(D)** DI and **(E)** LD on citrus fruits after 20, 23, and 26 days of inoculation at 4°C. The cells harvested from NYDB medium without exposure to H_2_O_2_ (I) and cells harvested from NYDB medium amended without (II) or with 1 mM proline (III) were exposed to 40 mM H_2_O_2_ for 90 min and then seeded to the wounds. Wounds inoculated with SDW followed by the pathogen were used as controls. Vertical bars represent standard errors of the mean. Data in columns with different letters within each parameter are significantly different according to Duncan’s multiple range test (*p* < 0.05).

As shown in Figure 8, *M*. *citriensis* effectively inhibited the growth of *P. digitatum* on citrus fruits at 25 and 4°C. The exogenous oxidative stress significantly reduced the biocontrol ability of *M*. *citriensis* at 25°C, but not at 4°C. On the 6th day after inoculation at 25°C, the DI value of citrus fruits treated with *M*. *citriensis* cells without exogenous oxidative stress (Treatment I) was 23%, whereas DI value of fruits treated with H_2_O_2_-exposure cells (Treatment II) was as high as 33%. However, there was no significant difference of the DI or the LD value of the fruits between Treatment I and Treatment II. Obviously, the biocontrol performance of proline-treated cells showed a significant increase. The DI value (10%) and the LD value (7 mm) of citrus fruits treated with proline-treated yeast cells (Treatment III) were significantly lower than those of citrus fruits in Treatment I and Treatment II stored at 25°C (Figures 8A–C). Moreover, compared with the fruits in Treatment I (43%), DI value of the fruits seed with proline-treated cells distinctly lower (30%) after 26 days of incubation at 4°C, while DI value of the control (inoculated with SDW instead of *M*. *citriensis* cells) reached 100% (Figure 8D).

### Biofilm Formation of *M. citriensis*

To investigate whether proline could promote the biofilm formation of *M*. *citriensis*, the yeast adherence on the polystyrene surface and the biofilm formation in citrus wounds by SEM were examined. Biofilms are microorganisms communities fixed to a surface and protected by EPS (Chmielewski and Frank, 2003).

**Figure 9:**
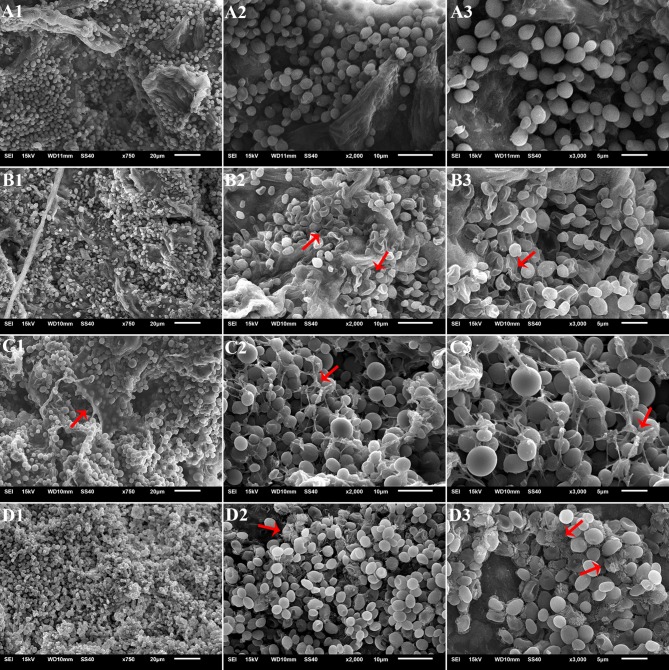
Scanning electron micrographs of biofilm formation of *Metschnikowia citriensis* in citrus wounds on the 26th day after inoculation at 4°C. The cells harvested from NYDB medium **(B1–B3)** and cells harvested from NYDB medium containing 1 mM proline **(C1–C3,D1–D3)** were exposed to 40 mM H_2_O_2_ for 90 min and then seeded to the wounds. Arrows show the cell depression **(B3)**, cell rupture **(B2)**, and extracellular matrix of biofilm **(C1–C3,D1–D3)**. The cells harvested from NYDB medium without exposure to H_2_O_2_ were used as controls **(A1–A3)**. Magnification of 750× **(A1–D1)**, 2000× **(A2–D2)**, and 3,000× **(A3–D3)**.

**Figure 10:**
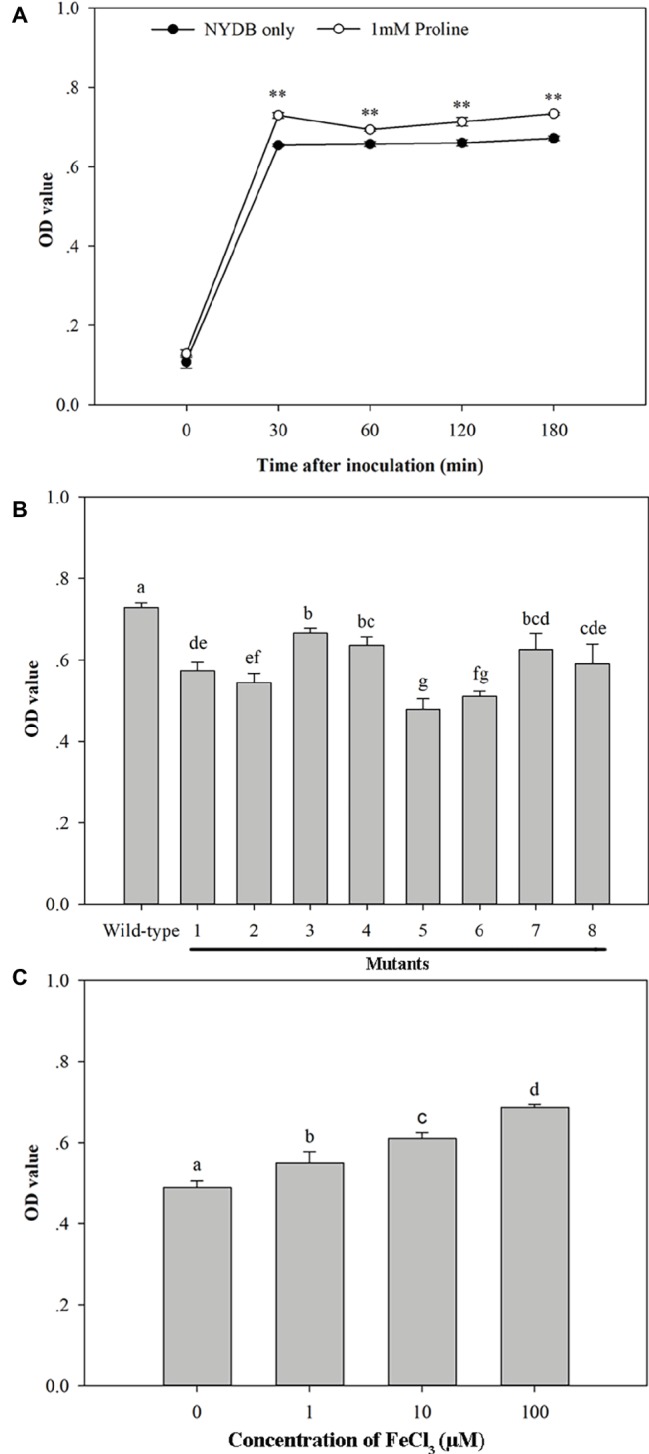
Biofilm formation ability of *Metschnikowia citriensis in vitro*. **(A)** Biofilm formation ability of wild-type *M*. *citriensis* FL01^T^ harvested from NYDB medium with or without proline. **(B)** Biofilm formation ability of wild-type and the low-pigment mutants of *M*. *citriensis* FL01^T^ harvested from NYDB medium. **(C)** Biofilm formation ability of wild-type *M*. *citriensis* FL01^T^ grew in YNB medium supplemented with different iron concentrations. The vertical bars represent standard errors of three replicates. Data with “**” and different letters within each parameter are significantly different according to the Duncan’s multiple range test (*p* < 0.01).

SEM documented biofilm formation in the surface of wounds (Figure 9). Exogenous oxidative stress showed little effect on the adhesion capability of *M*. *citriensis*, since the number of cells adhered onto each wound was similar to that of the control (the untreated cells without exposure to H_2_O_2_). However, after 26 days of storage at 4°C, untreated cells with exposure to H_2_O_2_ showed a greater number of depressed and even broken cells compared with the control (Figures 9A1,B1). *In vitro*, proline-treated cells had a high film-forming capacity after a 30-min incubation, since its OD value was significantly higher than that of the cells cultured in NYDB without proline (Figure 10A). Moreover, the proline-treated cells (Figures 9C1,D1) displayed even heavier cell clusters adhering to wounds after repeated washes, compared with the control. Proline increased EPS production of *M*. *citriensis*, and the biofilms of proline-treated cells displayed varied phenotypes, including somewhat flat biofilm structures with large pieces of mucus (Figures 9C1–C3) and more robust and thick biofilms with crystalline EPS (Figures 9D1–D3). These results suggested that exogenous oxidative stress contributed to harmful changes of the cell morphology at low temperature, but proline treatment could protect cells and promote the biofilm formation.

Similar to the effect of proline treatment, the pigment production (i.e., the pigmentation of the yeast cultures) and biofilm formation ability of *M*. *citriensis* that incubated in YNB supplemented with different concentrations of iron (0, 1, 10, 100 μM) increased with increasing iron concentration (Figure 10C). In contrast, the iron chelator tropolone reduced biofilm formation ability of *M*. *citriensis* (data not show). To investigate whether the pigment production of *M*. *citriensis* is related to its biofilm formation ability, cells of *M*. *citriensis* FL01^T^ were mutagenized with nitrosoguanidine. Eight pink mutants (with various degrees of pigmentation) that had less pigment production than the wild-type colonies (maroon) were selected, and the low-pigment mutants all showed lower capability of biofilm formation (Figure 10B), indicating that the pigment production promotes biofilm formation of *M*. *citriensis*.

## Discussion

In this study, the effect of proline on oxidative stress tolerance and biocontrol ability of *M*. *citriensis* and its possible mechanisms were investigated. We found that inducing pigment production with exogenous substances is a promising approach to enhance the biocontrol ability of *M*. *citriensis*.

*Metschnikowia* strains have been reported to release a diffusible colorless precursor and then the precursor immobilized iron to form a pigment, playing an important role in their antimicrobial activity (Sipiczki, 2006; Parafati et al., 2015). But it is different from the low molecular iron chelators released by other microorganisms to absorb iron in the environment, since this pigment is not soluble in water and observed to cover the outside of the cell, instead of accumulating in the cell (Kluyver et al., 1953). Moreover, the intracellular iron content of *M*. *citriensis* decreased with the increase of pigment production, suggesting that the proposed mechanism of releasing this pigment precursor is to prevent excessive accumulation of cellular iron.

Iron is relevant to intracellular ROS production and cell death. Iron and iron derivatives such as [Fe-S] clusters and heme are not only incorporated into ROS-producing enzymes but are also essential for their function, while superoxide and H_2_O_2_ can damage [4Fe-4S] clusters of proteins that lead to the release of Fe^2+^ (Dixon and Stockwell, 2014). The small pools (<20 μM) of Fe^2+^, which can directly catalyze the formation of destructive free radicals by Fenton chemistry [Fe^2+^ react with peroxides to form soluble hydroxyl (HO^•^) or lipid alkoxy (RO^•^)] and then result in damage to various biomolecules, are redox-active and reside in the mitochondrial matrix and the cytosol of eukaryotic cells (Petrat et al., 2002; Kell, 2009; Dixon and Stockwell, 2014). In addition to reducing intracellular iron levels by pigment formation, proline treatment induced a significant increase in SOD activity in the early stage and increased CAT activity in the later stage of oxidative stress to reduce ROS accumulation in *M*. *citriensis*. Similar to proline treatment, the tropolone-treated cells showed an early rise in SOD activity under oxidative stress compared with the control, although their SOD activity was elevated later than the proline-treated cells; tropolone treatment also increased CAT activity of *M*. *citriensis*, although their CAT activity was lower than that of proline-treated cells (data not shown). So, tropolone also reduced ROS levels of *M*. *citriensis* as a result of reduced iron content and regulation of antioxidant enzyme activity. SOD acts as the first line of defense against ROS in the cells, and it may be the core of the defense mechanism since its activity determines the concentrations of both $O_{2}^{•-}$ and H_2_O_2_ (Bowler et al., 1992; Blokhina et al., 2003). Although the reactivity of H_2_O_2_ is lower than $O_{2}^{•-}$, HO^•^ formation can occur in the Fenton reaction in biological systems when reduced transition metals such as Fe^2+^ are present (Blokhina et al., 2003). SOD and other enzymes, such as CAT and antioxidants, may have a highly optimized balance that works together to reduce the risk of HO^•^ formation; therefore, discussing the role of SOD requires the entire oxidant stress defense system to be considered as a whole (Bowler et al., 1992). Early rise of SOD activity of *M*. *citriensis* could accelerate the conversion of $O_{2}^{•-}$ to H_2_O_2_, and then the increase in CAT activity could promote the conversion of H_2_O_2_ to water.

Apoptosis is a major contributor to the cell viability loss in *M*. *citriensis* under oxidative stress, which is consistent with the results of other antagonistic yeasts in previous reports (Chen et al., 2015; Zhang et al., 2017). Proline opposed the ROS- and iron-induced apoptosis of *M*. *citriensis*, and the pigment induced by proline played an important role in inhibiting iron-dependent apoptotic cell death. Moreover, proline also prevented *M*. *citriensis* from other types of cell death associated with damage of plasma membranes, and proline suppressed which might because of the capability to stabilize cellular homeostasis during stress conditions (Szabados and Savoure, 2010). Therefore, the viability of *M*. *citriensis* was greatly increased by proline under oxidative stress.

Exposure of *M. citriensis* to exogenous H_2_O_2_ reduced its ability to grow and survive in wounds at 25 and 4°C and significantly reduced biocontrol efficacy of *M*. *citriensis* at 25°C, but not at 4°C, suggesting the effect of population density on its biocontrol efficacy at 4°C is less than that at 25°C. Although oxidative stress reduced the growth ability of *M*. *citriensis*, proline-treated cells with exogenous oxidative stress showed better biocontrol efficacy than the untreated cells with exogenous oxidative stress and the control (untreated cells without exogenous oxidative stress) not only at 25°C but also at 4°C, because proline increased pigment production and biofilm formation of *M. citriensis*. Biofilms generally exist in harsh environmental conditions (Fuqua et al., 1994; Rendueles and Ghigo, 2015), cells in which exhibit increased tolerance to harsh conditions (Whiteley et al., 2001; Harriott and Noverr, 2009; Bridier et al., 2011; Pu et al., 2014). As well as stress tolerance, biocontrol agent biofilms are frequently associated with spatial competition with pathogens, quickly forming layers on the surface of the nutrients and completely saturating the interface with the nutrients to limit nutrients into the pathogen (Habimana et al., 2011). Furthermore, since the presence of EPS in biofilms can locally concentrate the antimicrobial secondary metabolites produced by antagonists (Pandin et al., 2017), *M*. *citriensis* cells in biofilms can form higher concentrations of pigment relative to cells in solitary or planktonic forms. Cells in biofilms also exhibit an altered phenotype in growth rate (Harding et al., 2009). We found that the rate of cell growth of proline-treated cells with H_2_O_2_-exposure early in the storage was slower than that of the control and even slower than the untreated cells with H_2_O_2_-exposure when incubated at 4°C. Such behavior might imply that proline-treated yeast cells were preparing for the switch early in the storage, from growth to adaptation to environmental changes, because the physiological mechanisms of resistance to stress are likely to consume energy for its growth, including polymeric extracellular matrix production and biofilm formation. However, after cells are embedded in a self-secreted EPS, they can benefit from enhanced cell-to-cell communication and protect from stresses and efficient capture of nutrients, thereby promoting their growth (Harding et al., 2009). For this reason, proline enhanced the growth ability of *M. citriensis* late in the storage might be attributed to an increase in biofilm formation. It seems that the biofilm formation ability, which is one of the key biocontrol mechanisms of *M*. *citriensis*, is more important for its biocontrol efficacy, compared with the ability to grow rapidly in wounds.

Microorganisms in biofilms can use specific autoinducers that are signalling molecules allowing them to communicate with the surrounding populations, and these molecules are involved in the formation and development of biofilms (Fuqua et al., 1994; Rendueles and Ghigo, 2015). A vast number of natural signalling molecules regulate this quorum sensing (QS), especially the 2,5-diketopiperazines, and cyclodipeptides (CDPs) that belong to the non-ribosomal peptides and consist of two amino acids linked by peptide bonds (Bonnefond et al., 2011; Seguin et al., 2011; González et al., 2017). Because the intermediates cyclo-L-leucyl-L-leucyl (CDP) and pulcherriminic acid are produced during the formation of the pigment (pulcherrimin) of *Metschnikowia* from leucine (MacDonald, 1965), a hypothesis is that one of these intermediates might act as a diffusible signal factor to modulate QS and biofilm formation, but purified intermediates for this activity have not been tested. Since increased pigment production was accompanied by an increase in biofilm formation ability of *M*. *citriensis* and the low-pigment mutants of *M*. *citriensis* FL01^T^ all showed lower capability of biofilm formation, pigment production could promote the biofilm formation of *M*. *citriensis*. The pigment production might be a mechanism by which *M*. *citriensis* promotes protective biofilm formation against stress conditions.

## Conclusion

The results indicated that the application of proline was a useful approach to diminish the intracellular ROS level and enhance the oxidative stress tolerance of *M. citriensis* by regulating CAT and SOD enzyme activities and reducing intracellular iron content. Moreover, because of the increased pigment production and the robust biofilm formation in wounds, adding proline significantly improved biocontrol efficacy of *M. citriensis* against *P. digitatum* in citrus fruits. Pigment production contributed to the reduction of intracellular iron content and the biofilm formation of *M. citriensis*.

## Data Availability

All datasets generated for this study are included in the manuscript.

## Author Contributions

YL, CR, LD, and KZ designed the study. YL and SY conducted the experiments. YL, LY, and KZ wrote the manuscript. All authors have read and approved the submitted version.

### Conflict of Interest Statement

The authors declare that the research was conducted in the absence of any commercial or financial relationships that could be construed as a potential conflict of interest.
